# The association between varying levels of palliative care involvement on costs during terminal hospitalizations in Canada from 2012 to 2015

**DOI:** 10.1186/s12913-021-06335-1

**Published:** 2021-04-13

**Authors:** Sarina R. Isenberg, Christopher Meaney, Peter May, Peter Tanuseputro, Kieran Quinn, Danial Qureshi, Stephanie Saunders, Colleen Webber, Hsien Seow, James Downar, Thomas J. Smith, Amna Husain, Peter G. Lawlor, Rob Fowler, Julie Lachance, Kimberlyn McGrail, Amy T. Hsu

**Affiliations:** 1 Bruyère Research Institute, 43 Bruyère St, Office 264J-G, Ottawa, ON K1N 5C8 Canada; 2 Department of Family and Community Medicine, University of Toronto, Toronto, Canada; 3 Institute for Health Policy Management and Evaluation, University of Toronto, Toronto, Canada; 4 Department of Medicine, University of Ottawa, Ottawa, Canada; 5 Centre for Health Policy and Management, Trinity College Dublin, Dublin, Ireland; 6 The Irish Longitudinal study on Ageing (TILDA), Trinity College Dublin, Dublin, Ireland; 7 Division of Palliative Care, Department of Medicine, University of Ottawa, Ottawa, Canada; 8 Ottawa Hospital Research Institute, Ottawa, Canada; 9 Department of Medicine, University of Toronto, Toronto, Canada; 10 Department of Medicine, Division of Internal Medicine, Sinai Health, Toronto, Canada; 11 Temmy Latner Centre for Palliative Care, Sinai Health, Toronto, Canada; 12 Department of Oncology, McMaster University, Hamilton, Canada; 13 Department of Medicine, Johns Hopkins Hospital and Health System, Baltimore, USA; 14 Department of Oncology, Johns Hopkins Hospital and Health System, Baltimore, USA; 15 Interdepartmental Division of Critical Care Medicine, University of Toronto, Toronto, Canada; 16 Tory Trauma Program, Sunnybrook Hospital, Toronto, Canada; 17 End-of-Life Care Unit, Strategic Policy Branch, Health Canada, Ottawa, Canada; 18 Centre for Health Services and Policy Research, School of Population and Public Health, The University of British Columbia, Vancouver, Canada

**Keywords:** Palliative care, Acute care costs, Terminal hospitalizations, End of life

## Abstract

**Background:**

Inpatient palliative care is associated with lower inpatient costs; however, this has yet to be studied using a more nuanced, multi-tiered measure of inpatient palliative care and a national population-representative dataset. Using a population-based cohort of Canadians who died in hospital, our objectives were to: describe patients’ receipt of palliative care and active interventions in their terminal hospitalization; and examine the relationship between inpatient palliative care and hospitalization costs.

**Methods:**

Retrospective cohort study using data from the Discharge Abstract Database in Canada between fiscal years 2012 and 2015. The cohort were Canadian adults (age ≥ 18 years) who died in hospital between April 1st, 2012 and March 31st, 2015 (*N* = 250,640). The exposure was level of palliative care involvement defined as: medium-high, low, or no palliative care. The main measure was acute care costs calculated using resource intensity weights multiplied by the cost of standard hospital stay, represented in 2014 Canadian dollars (CAD). Descriptive statistics were represented as median (IQR), and n(%). We modelled cost as a function of palliative care using a gamma generalized estimating equation (GEE) model, accounting for clustering by hospital.

**Results:**

There were 250,640 adults who died in hospital. Mean age was 76 (SD 14), 47% were female. The most common comorbidities were: metastatic cancer (21%), heart failure (21%), and chronic obstructive pulmonary disease (16%). Of the decedents, 95,450 (38%) had no palliative care involvement, 98,849 (38%) received low involvement, and 60,341 (24%) received medium to high involvement. Controlling for age, sex, province and predicted hospital mortality risk at admission, the cost per day of a terminal hospitalization was: $1359 (95% CI 1323: 1397) (no involvement), $1175 (95% CI 1146: 1206) (low involvement), and $744 (95% CI 728: 760) (medium-high involvement).

**Conclusions:**

Increased involvement of palliative care was associated with lower costs. Future research should explore whether this relationship holds for non-terminal hospitalizations, and whether palliative care in other settings impacts inpatient costs.

**Supplementary Information:**

The online version contains supplementary material available at 10.1186/s12913-021-06335-1.

## Background

High proportions of deaths in hospitals are an issue confronting several countries. In 2012 in Belgium, Canada, England, Germany, and Norway, the proportion of decedents who died in acute care hospitals ranged from 42 to 52% [1]. In 2018, 59.5% of all deaths in Canada occurred in hospital [2]. These terminal hospitalizations (i.e., hospitalizations wherein a patient dies) are costly. According to a study of Ontario decedents, $4.7 billion CAD was spent on health care in the last year of life and 42.9% of costs are derived from inpatient costs (an average of $30,872 CAD per person) [3]. Focusing on the last 180 days of life for patients with cancer, Canada had the highest per capita hospital expenditures at $21,840 USD, compared to the United States, Norway, Germany, Belgium, and the Netherlands [1].

Treatments that tend to drive these end-of-life costs are sometimes termed aggressive interventions, such as admission to intensive care units (ICU), mechanical ventilation, defibrillation, dialysis, percutaneous coronary interventions, and percutaneous feeding tube insertions. A recent study of decedents in Ontario demonstrated that, in the last 6 months of life, 19.4% were admitted to the ICU, and 13.9% received mechanical ventilation [4].

A meta-analysis on the association between palliative care consultations and direct hospital costs for adults with serious illness found that patients who received palliative care consultations had a statistically significant reduction in direct hospital costs (−$3237 USD; 95% CI, −$3581 to -$2893; *P* < .001) [5]. There are limitations to previous studies. Most studies focus on individual hospital or state-level costs. Most previous studies analyzed the relationship between palliative care and costs as a binary variable [6–9]. In reality there are differing levels of inpatient palliative care involvement. A binary variable does not give a good sense of how much palliative care people are receiving, and a more nuanced variable can help decision makers determine how to appropriately allocate resources.

Due to past studies’ lack of nuanced palliative care variables and lack of national scope, we sought to understand the relationship between differing levels of inpatient palliative care and hospitalization costs in terminal hospitalizations. Our first objective was to describe patients who died in hospital, and their receipt of different levels of palliative care and active interventions. Our second objective was to examine the relationship between terminal hospitalizations with varying levels of palliative care involvement and costs.

## Methods

### Study design, setting, and data sources

We conducted a national retrospective cohort study, using patient-level health administrative data from the Discharge Abstract Database (DAD) in Canada (excluding the province of Quebec^1^ and the territories). The DAD is a national database that includes data from acute care institutions across Canada. It is mandatory for all provinces, except Quebec, to submit a DAD abstract for each hospital discharge. A trained Canadian Institute for Health Information data abstractor reviews the medical record for the encounter and makes determinations about the care received to populate the DAD. Analysis was conducted from January 2020–July 2020.

### Study cohort

Our cohort included all adults who died in hospital between April 1st, 2012 and March 31st, 2015. We excluded people who were under age 18 or over age 105 at death, or who were non-Canadian residents. We excluded people with sex not identified as either male or female because there were few records and we could not meaningfully analyze the end-of-life experience of these individuals. We excluded people with missing postal code data, missing admission/discharge dates, or missing/zero inpatient resource intensity weight data, which results in missing/zero cost data. We excluded people from Yukon, Northwest Territories, and Nunavut due to small sample sizes and data unavailable for costs of a standard hospital stay in the Northwest Territories and Nunavut. We also excluded people with erroneous data entries concerning their admissions and death (e.g., multiple terminal admissions). Finally, we excluded people whose terminal hospitalization had a length of stay greater than 180 days, as these people are not representative of a typical acute care stay and may be capturing long-term care beds that exist within some Canadian hospitals.

### Patient characteristics

We measured demographic and clinical variables including age, sex, rurality, province, comorbidities and chronic conditions defined as per the Elixhauser index [10] and the Van Walraven index (a weighted numeric score based on the Elixhauser classification system designed to predict in-hospital mortality) [11], represented as empirical quartiles of the score observed in our sample. We also measured ICU stay, receipt of major surgery [12] and “any active intervention”, defined as mechanical ventilation, cardiopulmonary resuscitation (CPR), defibrillation, dialysis, percutaneous coronary intervention, percutaneous feeding tube insertion, blood transfusion, and bronchoscopy. We developed this list of active interventions based on consensus among our group of experts in palliative and intensive care.

### Exposure

Our primary exposure was palliative care involvement, adapted from Webber et al. [13]. For each admission, the DAD captures up to 25 diagnosis codes based on the 10th revision of the International Statistical Classification of Diseases and Related Health Problems (ICD-10). Medium-high palliative involvement was defined as a hospitalization with palliative care as the most responsible service provider (i.e., the service that treated the patient for the majority of their hospital stay; this could include care delivered by a specialist palliative care provider or a generalist providing palliative care) and/or the most responsible diagnosis (i.e., when the patient is admitted solely to receive palliative care or when the patient received palliative care prior to hospital arrival but was not admitted for a reversible cause; using ICD-10 diagnosis code Z51.5). Low Palliative involvement was defined as a hospitalization with a secondary diagnosis of palliative care (i.e., using diagnosis codes 2 through 25), excluding most responsible provider. No involvement was defined as a hospitalization without a palliative diagnosis or most responsible service provider. In Canada, inpatient palliative care is largely provided as a consult service; here palliative care would not be noted as most responsible provider, but would likely be noted as most responsible diagnosis or secondary diagnosis. Inpatient palliative care might also include palliative care units, which would have the palliative care designation as most responsible provider.

### Outcome

The total costs of a terminal hospitalization were calculated using validated costing methods [14–17]. Each acute care hospital admission is associated with an inpatient resource intensity weight (RIW), which measures the intensity of resource use associated with medical, diagnostic and surgical procedures, adjusted for patient and institutional characteristics (e.g., case-mix adjustments). Inpatient hospital costs are calculated by multiplying the RIWs by a cost-of-standard-hospital-stay, which is measured at the provincial level. We also adjusted costs to 2014 using the Statistics Canada Health Care Consumer Price Index (CPI) [18]. All costs are in Canadian dollars.

### Statistical analysis

For Objective 1, wherein we describe patients who died in hospital and their receipt of palliative care, we presented descriptive statistics as mean (standard deviation (SD)), median (interquartile range (IQR)), and n(%). For Objective 2, wherein we examined the relationship between varying levels of palliative care involvement and costs, we fit a generalized estimating equation (GEE) model, assuming our response variable (inpatient hospital costs) followed a gamma distribution. We used a log link function. We accounted for clustering by hospital using a compound symmetric working correlation structure. We include the natural logarithm of patient length of stay as an offset in our gamma GEE models. Our unadjusted model had palliative care involvement as the only predictor and costs as the outcome. Based on clinical expertise and estimated bivariate associations from our unadjusted models, we built a multivariable gamma GEE model controlling for age, sex, province, and Van Walraven index. We present regression coefficient estimates (representing log cost-ratios), 95% confidence intervals (CIs) and *p*-values from the fitted gamma GEE models. For both unadjusted and adjusted models, we present the conditional expected value of cost (and its 95% CI) at each level of palliative care intensity, controlling for other variables in the model.

We conducted two sensitivity analyses. First, we estimated stratified multivariable models by province because palliative care delivery [19] and the cost of a standard hospital stay differs across provinces. Second, we estimated stratified multivariable models by Elixhauser comorbidity categories [10], specifically metastatic cancer, heart failure, major neurocognitive disorders, chronic obstructive pulmonary disease (COPD), kidney failure, human immunodeficiency virus/acquired immunodeficiency syndrome (HIV/AIDS), and liver failure. We focused on these diseases categories as they are known to play a role in the relationship between palliative care receipt and associated costs [5, 20]. We report adjusted costs from the stratified gamma GEE model fits. All analyses were performed using SAS version 9.4 (SAS Institute, Cary, North Carolina).

This study was approved by Institutional Ethics Review Boards at Sinai Health and the Ottawa Health Services Network. While data sharing agreements prohibit the research team and Canadian Institute for Health Information from making the dataset publicly available, access may be granted to those who meet pre-specified criteria for confidential access. The underlying analytic code are available from the authors upon request, with the understanding that the programs may rely on coding templates or macros that are unique to Canadian Institute for Health Information.

## Results

### Baseline characteristics

We identified 290,855 adult hospital decedents. We excluded 40,215 adults, resulting in a cohort of 250,640 people (see Supplementary Appendix for a full list of exclusions). Of the decedents, 95,450 (38%) had no inpatient palliative care involvement, 98,849 (38%) received low palliative care involvement, and 60,341 (24%) received medium-high palliative care involvement. Mean age was 76 years (SD = 14 years), 47% were female, 47% were from Ontario, and 26% received any active intervention (Table 1). The most common comorbidities were: metastatic cancer (21%), heart failure (21%), and COPD (16%). Table 2 displays the terminal hospitalization episode costs per day according to patient characteristics. Costs decreased as level of palliative care involvement increased. There was large provincial variation in mean cost of terminal hospitalization episode with the highest median costs per day being in Alberta at $1250 (IQR 1022–1684) and the lowest median costs being in New Brunswick at $797 (IQR 657–1015). Median costs were higher among patients who received any active intervention at $2088 (IQR 1396–2981) compared to those who received no active intervention at $881 (IQR 730–1074). The Supplementary Appendix displays a table demonstrating the relationship between level of palliative care involvement, daily costs, and length of stay; as level of palliative care involvement increases, cost per day decreases, and as length of stay increases, costs decrease.

**Table 1:** Patient demographic and clinical characteristics stratified by level of palliative care involvement

Category	n (***n*** = 250,640)	No involvement n(%) (***n*** = 95,450)	Low involvement n(%) (***n*** = 98,849)	Medium-high involvement n(%) (***n*** = 60,341)
Age				
18–40	4401	2280 (2)	1382 (2)	739 (1)
41–55	16,778	6157 (6)	5948 (6)	4673 (8)
56–70	54,974	18,999 (20)	20,248 (21)	15,727 (26)
71–85	104,250	39,738 (42)	40,139 (42)	24,373 (40)
86–105	70,237	28,276 (30)	27,132 (29)	14,829 (25)
Sex				
F	118,534	43,364 (45)	45,211 (48)	29,959 (50)
Rurality				
Yes	49,563	20,286 (21)	15,965 (17)	13,312 (22)
Province				
AB	29,562	12,591 (13)	10,869 (11)	6102 (10)
BC	41,283	16,333 (17)	16,103 (17)	8847 (15)
MB	15,244	4776 (5)	5654 (6)	4814 (8)
NB	10,992	3435 (4)	3920 (4)	3637 (6)
NL	7199	2499 (3)	2388 (3)	2312 (4)
NS	13,494	3271 (3)	4936 (5)	5287 (9)
ON	118,410	46,639 (49)	46,200 (49)	25,571 (42)
PE	1856	675 (1)	696 (1)	485 (1)
SK	12,600	5231 (5)	4083 (4)	3286 (5)
Number of comorbidities				
0	25,186	12,807 (13)	8176 (9)	4203 (7)
1	84,879	26,569 (28)	28,184 (30)	30,126 (50)
2	64,604	24,015 (25)	25,842 (27)	14,747 (24)
3+	75,971	32,059 (34)	32,647 (34)	11,265 (19)
Hospital length of stay (days)				
0–2	56,892	28,992 (30)	13,294 (14)	14,606 (24)
3–7	64,081	25,527 (27)	22,905 (24)	15,649 (26)
8–19	66,797	21,987 (23)	29,571 (31)	15,239 (25)
20+	62,870	18,944 (20)	29,079 (31)	14,847 (25)
Heart failure	52,510	25,076 (26)	21,711 (23)	5723 (9)
COPD	39,731	17,240 (18)	16,541 (17)	5950 (10)
HIV	418	181 (0)	170 (0)	67 (0)
Liver failure	16,096	6295 (7)	7457 (8)	2344 (4)
Metastatic cancer	52,734	6992 (7)	19,475 (21)	26,267 (44)
Major neurocognitive disorders	19,249	7393 (8)	8545 (9)	3311 (5)
Kidney failure	23,130	10,042 (11)	9749 (10)	3339 (6)
Van Walraven Index				
Quartile −1-3	58,870	28,693 (30)	19,747 (21)	10,430 (17)
Quartile 4–8	65,252	27,308 (29)	23,490 (25)	14,454 (24)
Quartile 9–15	60,678	23,967 (25)	24,750 (26)	11,961 (20)
Quartile 16+	65,840	15,482 (16)	26,862 (28)	23,496 (39)
Major Surgery^a^	47,068	23,218 (24)	20,825 (22)	3025 (5)
ICU stay	75,302	39,774 (42)	31,117 (33)	4411 (7)
Mechanical ventilation	52,022	29,487 (31)	20,288 (21)	2247 (4)
CPR	13,656	10,920 (11)	2551 (3)	185 (0)
Defibrillation	5172	3789 (4)	1293 (1)	90 (0)
Dialysis	11,860	6102 (6)	5179 (5)	579 (1)
Percutaneous Coronary Intervention	2616	1865 (2)	690 (1)	61 (0)
Feeding tube	6626	2956 (3)	2993 (3)	677 (1)
Blood Transfusion	201	96 (0)	81 (0)	24 (0)
Bronchoscopy	3264	1633 (2)	1469 (2)	162 (0)
Any active intervention^b^	64,897	36,726 (38)	24,729 (26)	3442 (6)

*Abbreviations*: *F* Female, *AB* Alberta, *BC* British Columbia, *MB* Manitoba, *NB* New Brunswick, *NL* Newfoundland, *NS* Nova Scotia, *ON* Ontario, *PE* Prince Edward Island, *SK* Saskatchewan, *COPD* Chronic obstructive pulmonary disease, *HIV* Human immunodeficiency virus, *ICU* Intensive care unit, *CPR* Cardiopulmonary resuscitation

^a^Major surgery is defined according to anatomic site: abdominal, cardiac, retroperitoneal, thoracic and vascular [12]

^b^Any active intervention is defined as any of the following: mechanical ventilation, CPR, defibrillation, dialysis, percutaneous coronary intervention, feeding tube, blood transfusion, and bronchoscopy

**Table 2:** Costs per day of a terminal hospitalizations according to patient demographic and clinical characteristics

Category	n	Costs median (IQR)
Palliative care involvement		
No involvement	95,450	1154 (888–1882)
Low involvement	94,849	1032 (856–1486)
Medium-high involvement	60,341	726 (661–875)
Age		
18–40	4401	1834 (1040–2974)
41–55	16,778	1186 (818–2221)
56–70	54,974	1050 (779–1803)
71–85	104,250	981 (783–1419)
85+	70,237	911 (768–1139)
Sex		
F	118,534	957 (771–1352)
M	132,106	1001 (790–1520)
Rurality		
Non-rural	201,077	985 (781–1464)
Rural	49,563	955 (774–1317)
Province		
AB	29,562	1250 (1022–1684)
BC	41,283	947 (770–1303)
MB	15,244	911 (748–1225)
NB	10,992	797 (657–1015)
NL	7199	957 (774–1268)
NS	13,494	877 (718–1145)
ON	118,410	949 (761–1481)
PE	1856	919 (757–1161)
SK	12,600	1103 (897–1504)
Number of comorbidities		
0	25,186	1073 (823–1784)
1	84,879	894 (717–1228)
2	64,604	975 (787–1408)
3+	75,971	1054 (848–1582)
Heart failure		
No	198,130	961 (758–1433)
Yes	52,510	1031 (863–1446)
COPD		
No	210,909	966 (769–1433)
Yes	39,731	1024 (849–1444)
HIV		
No	250,222	977 (780–1434)
Yes	418	1440 (1015–2344)
Liver failure		
No	234,544	966 (774–1393)
Yes	16,096	1220 (907–2091)
Metastatic cancer		
No	197,906	1037 (824–1588)
Yes	52,734	815 (694–1033)
Major neurocognitive disorders		
No	231,391	967 (775–1384)
Yes	19,249	1207 (857–2247)
Kidney failure		
No	227,510	972 (773–1435)
Yes	23,130	1031 (845–1440)
Van Walraven Index		
Quartile −14-3	58,870	1047 (816–1636)
Quartile 4–8	65,252	991 (786–1496)
Quartile 9–15	60,678	1010 (819–1473)
Quartile 16+	65,840	897 (726–1197)
Hospital Length of Stay		
0–3	56,892	1062 (745–1643)
4–7	64,081	985 (823–1371)
8–20	66,797	972 (805–1389)
30+	62,870	929 (747–1325)
ICU stay		
No	175,338	877 (727–1077)
Yes	75,302	1808 (1146–2788)
Mechanical ventilation		
No	198,618	895 (740–1103)
Yes	52,022	2350 (1647–3151)
CPR		
No	236,984	956 (770–1343)
Yes	13,656	2009 (1366–2926)
Defibrillation		
No	245,468	969 (776–1394)
Yes	5172	2310 (1508–3352)
Dialysis		
No	238,780	960 (771–1359)
Yes	11,860	2035 (1294–3042)
Percutaneous Coronary Intervention		
No	248,024	973 (778–1409)
Yes	2616	3611 (2560–5087)
Feeding tube		
No	244,014	969 (775–1402)
Yes	6626	1609 (1114–2577)
Blood transfusion		
No	250,439	978 (780–1435)
Yes	201	1580 (1004–2949)
Bronchoscopy		
No	247,376	972 (777–1406)
Yes	3264	2604 (1757–3206)
Any active intervention^a^		
No	185,743	881 (730–1074)
Yes	64,897	2088 (1396–2981)
Major surgery^b^		
No	203,572	902 (744–1134)
Yes	47,068	2046 (1435–2999)

*Abbreviations*: *F* Female, *M* Male, *AB* Alberta, *BC* British Columbia, *MB* Manitoba, *NB* New Brunswick, *NL* Newfoundland, *NS* Nova Scotia, *ON* Ontario, *PE* Prince Edward Island, *SK* Saskatchewan, *COPD* Chronic obstructive pulmonary disease, *HIV* Human immunodeficiency virus, *ICU* Intensive care unit, *CPR* Cardiopulmonary resuscitation

^a^Any active intervention is defined as any of the following: mechanical ventilation, CPR, defibrillation, dialysis, percutaneous coronary intervention, feeding tube, blood transfusion, and bronchoscopy

^b^Major surgery is defined according to anatomic site: abdominal, cardiac, retroperitoneal, thoracic and vascular [12]

### Main analyses

In the unadjusted analysis, compared to no palliative care involvement, palliative care involvement was associated with lower costs per hospital-day (Table 3). At no involvement (i.e., the reference), the mean cost per hospital-day was $1414 (95% CI 1377: 1453). At low involvement, the cost was $1180 (95% CI 1155: 1206). At medium-high involvement, the cost was $737 (95% CI 721: 753).

**Table 3:** Regression coefficient estimates (on log cost-ratio scale^a^), 95% confidence intervals and *p*-values from unadjusted and adjusted gamma Generalized Estimating Equation models for cost per day outcome

Category	Unadjusted Models^b^		Adjusted Model^c^	
	Coefficient (95% CI)	*P*-value	Coefficient (95% CI)	*P*-value
Palliative Care Intensity				
No involvement	Ref		Ref	
Low involvement	−0.18 (− 0.21: − 0.15)	< 0.001	− 0.15 (− 0.17: − 0.12)	< 0.001
Medium involvement	− 0.65 (− 0.69: − 0.62)	< 0.001	− 0.6 (− 0.63: − 0.57)	< 0.001
Age				
18–40	Ref		Ref	
41–55	− 0.22 (− 0.27: − 0.18)	< 0.001	− 0.15 (− 0.18: − 0.11)	< 0.001
56–70	−0.32 (− 0.36: − 0.27)	< 0.001	−0.23 (− 0.27: − 0.19)	< 0.001
71–85	−0.45 (− 0.5: − 0.39)	< 0.001	−0.38 (− 0.42: − 0.34)	< 0.001
86–104	−0.64 (− 0.7: − 0.59)	< 0.001	−0.58 (− 0.62: − 0.54)	< 0.001
Sex				
F	Ref		Ref	
M	0.08 (0.07: 0.09)	< 0.001	0.04 (0.03: 0.05)	< 0.001
Province				
ON	Ref		Ref	
AB	−0.02 (−0.19: 0.14)	0.79	0.02 (−0.07: 0.11)	0.66
BC	0.02 (−0.1: 0.13)	0.78	0.03 (−0.05: 0.11)	0.48
MB	−0.02 (− 0.14: 0.11)	0.81	0 (− 0.08: 0.09)	0.96
NB	−0.02 (− 0.35: 0.31)	0.91	− 0.01 (− 0.24: 0.21)	0.9
NL	0.05 (−0.1: 0.2)	0.52	0.04 (−0.06: 0.13)	0.44
NS	−0.19 (− 0.61: 0.24)	0.39	− 0.11 (− 0.37: 0.15)	0.4
PE	0.48 (0.17: 0.8)	0.002	0.39 (0.09: 0.69)	0.01
SK	0.06 (−0.03: 0.14)	0.23	0.07 (0.01: 0.13)	0.02
Van Walraven Index				
−14-3	Ref		Ref	
4–8	−0.07 (−0.08: − 0.05)	< 0.001	− 0.04 (− 0.05: − 0.03)	< 0.001
9–15	− 0.1 (− 0.12: − 0.08)	< 0.001	− 0.09 (− 0.1: − 0.07)	< 0.001
16+	− 0.25 (− 0.28: − 0.22)	< 0.001	− 0.2 (− 0.22: − 0.17)	< 0.001

*Abbreviations*: *F* Female, *AB* Alberta, *BC* British Columbia, *MB* Manitoba, *NB* New Brunswick, *NL* Newfoundland, *NS* Nova Scotia, *ON* Ontario, *PE* Prince Edward Island, *SK* Saskatchewan

^a^As explained in previous work, these “coefficient estimates [are] derived from generalized linear models with logarithmic link functions [and] are interpretable as the logarithm of the relative change in mean cost associated with a one-unit change in the predictor variable” (Austin PC, Ghali WA, Tu JV. A comparison of several regression models for analysing cost of CABG surgery. Stat Med. 2003 Sep 15;22 (17):2799–815. doi: 10.1002/sim.1442. PMID: 12939787)

^b^This column displays unadjusted models for each variable

^c^Model adjusted for age, sex, province, and Van Walraven index

After adjusting for age, sex, province, and Van Walraven index, increasing levels of palliative care involvement were incrementally associated with lower costs, compared to no palliative care involvement (Table 3). Among decedents with no involvement (i.e., the reference), the cost per hospital day was $1359 (95% CI 1323: 1397). At low involvement, the cost was $1175 (95% CI 1146: 1206). At medium-high involvement, the cost was $744 (95% CI 728: 760).

### Sensitivity analyses

Adjusting for age, sex, and Van Walraven index, we found that higher palliative care involvement was associated with lower costs in all provinces (Table 4) Adjusting for age, sex, province, and Van Walraven index, we found that higher palliative care involvement was associated with lower costs across Elixhauser comorbidity categories (Table 5).

**Table 4:** Estimated costs per day and 95% confidence intervals from adjusted^a^ gamma Generalized Estimating Equation (GEE) model, stratified by province

Province	Palliative care involvement	n	Costs (95% CI)
Alberta	No involvement	12,591	1563 (1490: 1640)
	Low involvement	10,869	1383 (1331: 1439)
	Medium-high involvement	6102	923 (882: 966)
British Columbia	No involvement	16,333	1400 (1321: 1484)
	Low involvement	16,103	1224 (1170: 1281)
	Medium-high involvement	8847	774 (738: 812)
Manitoba	No involvement	4776	1288 (1180: 1405)
	Low involvement	5654	1247 (1152: 1349)
	Medium-high involvement	4814	772 (700: 852)
New Brunswick	No involvement	3435	1324 (1173: 1493)
	Low involvement	3920	1007 (919: 1104)
	Medium-high involvement	3637	710 (657: 768)
Newfoundland	No involvement	2499	1447 (1318: 1590)
	Low involvement	2388	1206 (1120: 1299)
	Medium-high involvement	2312	826 (782: 873)
Nova Scotia	No involvement	3271	1386 (1234: 1557)
	Low involvement	4936	1248 (1141: 1366)
	Medium-high involvement	5287	755 (693: 823)
Ontario	No involvement	46,639	1394 (1336: 1454)
	Low involvement	46,200	1181 (1139: 1225)
	Medium-high involvement	25,571	726 (701: 752)
PEI	No involvement	675	1694 (1268: 2264)
	Low involvement	696	1420 (1082: 1863)
	Medium-high involvement	485	1029 (779: 1359)
Saskatchewan	No involvement	5231	1537 (1435: 1646)
	Low involvement	4083	1383 (1328: 1439)
	Medium-high involvement	3286	898 (852: 947)

^a^Model adjusted for age, sex, van Walraven index

**Table 5:** Estimated costs per day and 95% confidence intervals from adjusted^a^ gamma Generalized Estimating Equation model, stratified by Elixhauser disease condition

Disease	Palliative Care Intensity	n	Cost (95% CI)
Metastatic cancer	No involvement	6992	1204 (1172: 1237)
	Low involvement	19,475	1038 (1021: 1055)
	Medium-high involvement	26,267	754 (746: 762)
Heart failure	No involvement	25,076	1275 (1248: 1303)
	Low involvement	21,711	1178 (1158: 1198)
	Medium-high involvement	5723	814 (796: 832)
Major neurocognitive disorders	No involvement	7393	1578 (1530: 1628)
	Low involvement	8545	1448 (1408: 1489)
	Medium-high involvement	3311	835 (811: 859)
COPD	No involvement	17,240	1298 (1273: 1323)
	Low involvement	16,541	1246 (1223: 1269)
	Medium-high involvement	5950	789 (774: 804)
Kidney failure	No involvement	10,042	1307 (1278: 1336)
	Low involvement	9749	1201 (1179: 1224)
	Medium-high involvement	3339	829 (810: 849)
HIV	No involvement	181	1917 (1791: 2052)
	Low involvement	170	1817 (1684: 1961)
	Medium-high involvement	67	973 (857: 1104)
Liver Failure	No involvement	6295	1668 (1616: 1723)
	Low involvement	7457	1459 (1417: 1502)
	Medium-high involvement	2344	796 (768: 826)

^a^Model adjusted for age, sex, province, van Walraven index

## Discussion

In this study, we demonstrated increased involvement of palliative care was associated with lower costs.. However, a substantial proportion (38%) had no inpatient palliative care involvement in their terminal hospitalization and a minority (24%) received medium-high palliative care involvement. In addition to cost, these findings are also reflective of the care experience of Canadians who die in hospitals; our analysis found that the proportion of decedents with any active interventions decreased with increasing levels of palliative care involvement. This relationship between palliative care involvement and costs was sustained while stratifying across provinces and disease groups.

The relatively low level of inpatient palliative care receipt was also observed in a national study of decedents who were hospitalized in their last year of life, wherein 18.7% received inpatient palliative care consultation [21]. In our study, the majority of people received little to no palliative care in their terminal hospitalization. This finding is in line with our past work on terminal hospitalizations in Ontario from 2012 to 2015, which found that 36% never had access to palliative care [22]. These low levels of palliative care receipt are problematic, as the Canadian Institutes for Health Information estimates that 94% of patients who died in hospital were hospitalized for a non-sudden death condition that could potentially have benefitted from palliative care involvement [19]; however, it is unclear how that report operationalizes potential benefit.

Our findings reinforce past studies demonstrating that inpatient palliative care is associated with lower inpatient costs [5–9, 23]. A systematic review of the economic evidence on specialist palliative care consultations found that there were statistically significant savings of 9–25% associated with palliative care consultation [23]. However, ours is the first national study to use a tiered palliative care variable demonstrating the relationship between palliative care and lower costs. Importantly, our findings are restricted to terminal hospitalizations and future studies would need to analyze whether this relationship holds for other hospitalizations.

These lower costs associated with palliative care might be a consequence of patients receiving palliative care undergoing fewer active interventions and having fewer ICU stays, which is likely aligned with the patients’ underlying preferences for care. Other studies have found that palliative care consultations are associated with lower intensity of hospital treatment, less expensive procedures, shorter length of stay [23–30], decreased use of chemotherapy near death, and lower risk of ICU admission, multiple emergency department visits, and multiple hospitalizations near death [31]. Hsu et al.’s national, Canadian study found that, in an adjusted analysis, receipt of palliative care was associated with lower odds of ICU admission in the last 30 days of life (OR: 0.31, 95% CI: 0.30–0.32) [21]. Importantly, while palliative care is associated with a reduced likelihood of receipt of these treatments, it may be because patients’ preferences to receive less aggressive care is driving higher rates of palliative care and lower rates of these active interventions. Further, life-sustaining measures can cause or prolong suffering in a dying person, but the goals of palliative care and intensive care are not mutually exclusive. There are many models for integrating palliative care principles into the critical care environment [32], and one recent study found that among hospital decedents, ICU care was associated with higher family ratings of quality of end-of-life care than ward care [33].

The dose-response relationship between palliative care and costs was also observed across disease groups, including metastatic cancer, heart failure, major neurocognitive disorders, COPD, kidney failure, HIV/AIDS, and liver failure. These findings are consistent with disease-specific palliative care economic evaluations. A meta-analysis of six studies on the economic impact of inpatient palliative care consultations, found that for studies focusing on cancer, palliative care led to a reduction in costs (−$4251 USD; 95% CI, −$4664 to -$3837). The same study found that for noncancer populations, there was also an association between palliative care and lower costs (−$2105; 95% CI, −$2698 to -$1511) [5]. May et al. conducted a pooled analysis of four economic evaluations of non-cancer diseases. They found that inpatient palliative care was associated with a reduction in total direct costs for heart failure, neurodegenerative conditions, COPD, kidney failure, and liver failure. They did not find an association for HIV/AIDS [20].

The magnitude of costs varied across provinces, which is expected, as there are differences in provincial offerings of palliative care, and cost-of-standard-hospital-stay. However, some of these observed differences might be due to inter-provincial administrative coding differences. The mandatory elements to be coded vary by province, and there are noted inter-provincial inconsistencies in the format and coding of data elements, including diagnoses and procedures [34]. Notably, in our descriptive findings, the mean cost per day in Alberta was higher than any other province at $1250. This may relate to two factors, though further analysis is required to determine a causal link. First, Alberta has the highest per capita health spending of all the provinces, estimated at $7658 per person in 2019, compared to the national average at $7068 [35]. Second, Alberta has the lowest levels of inpatient palliative care of all the provinces at 15.2% [21]. When comparing people who died in acute care from 2016 to 2017, Alberta had the lowest percentage of people hospitalized primarily for palliative care (19%) [19]. Despite these variations, we observed that the dose-response relationship was maintained across provinces.

Our paper has several limitations. First, costs were derived in a top-down approach using resource intensity weights, which is less precise than a microcosting approach; thereby limiting the generalizability of our findings. Second, there may be inconsistencies with how often and under what circumstances palliative care is coded across the provinces. Challenges with the subjective nature of the deeming palliative care as the most responsible diagnosis code and the risk of this designation being overused or misused was noted by Downar et al. in 2010 [36]; however, these issues persist. Third, the method we used for identifying receipt of palliative care, approximates receipt of palliative care, but further research is needed to verify that these methods are truly identifying encounters with palliative care providers and that the designation of low or medium/high is appropriate. This method also does not capture the intensity of palliative care receipt. Fourth, there are two main limitations with using a decedent cohort in terminal hospitalizations. We cannot draw causal inferences; however, we can examine the costs of care provided at the end of life. Further physicians do not necessarily know when a patient is admitted to hospital that he/she will die in hospital, so our findings cannot necessarily be leveraged for prospective decision making. While prognostication is inexact, future prospective studies may more precisely be able to examine the relationship between palliative care involvement, hospitalizations, and costs. Fifth, there are limitations to using the DAD; we are unable to measure the frequency of palliative care (i.e., how many times a patient was seen by palliative care specialists) or the quality of palliative care, when in the hospitalization palliative care services were received, what constitutes a palliative care service or provider, and concordance between preferences for care and services received. While in our adjusted models we controlled for variables we thought might confound the relationship between palliative care and costs, there may still be uncontrolled confounding regarding who receives palliative care, as well as the availability of hospital-based, specialist palliative care. Finally, our data was restricted to inpatient data, which precluded us from assessing the impact that outpatient palliative care might have on our results. Outpatient palliative care has been associated with decreased health utilization and increased likelihood of home deaths [37–41], which might lead patients receiving outpatient palliative care to be excluded from our cohort.

## Conclusions

Increased palliative care involvement is associated with lower inpatient costs. This is the first national population-representative analysis on the impact of inpatient palliative care on costs using a tiered palliative care variable. Future research should explore whether this relationship holds for other hospitalizations towards the end of life, as well as whether palliative care in other settings impacts inpatient costs.

## Supplementary Information

**Additional file 1: Figure 1.** Cohort creation figure. **Table 1.** Median/IQR cost per day in each level of palliative involvement and hospital length of stay.

## Data Availability

The data that support the findings of this study are available from Canadian Institute for Health Information, but restrictions apply to the availability of these data, which were used under license for the current study, and so are not publicly available.

## Abbreviations

AB: Alberta
BC: British Columbia
CI: Confidence intervals
COPD: Chronic obstructive pulmonary disease
CPI: Consumer Price Index
CPR: Cardiopulmonary resuscitation
DAD: Discharge Abstract Database
F: Female
GEE: Generalized estimating equation
HIV/AIDS: Human immunodeficiency virus/acquired immunodeficiency syndrome
ICD: International Statistical Classification of Diseases and Related Health Problems
ICU: Intensive care unit
IQR: Interquartile range
MB: Manitoba
NB: New Brunswick
NL: Newfoundland
NS: Nova Scotia
ON: Ontario
PE: Prince Edward Island
SD: Standard deviation
SK: Saskatchewan

## Footnotes

1 Note: Quebec does not contribute to the DAD data collection.
