# Supplementary material for: The association between varying levels of palliative care involvement on costs during terminal hospitalizations in Canada from 2012 to 2015
Source: BMC Health Serv Res. 2021 Apr 13;21:331. doi: 10.1186/s12913-021-06335-1 (PMC8045222; doi:10.1186/s12913-021-06335-1)
Supplement: Supplementary file 1 — Additional file 1: Figure 1. Cohort creation figure. Table 1. Median/IQR cost per day in each level of palliative involvement and hospital length of stay. [file 12913_2021_6335_MOESM1_ESM.docx]

Supplementary appendix

Figure 1: Cohort creation figure^[[1]](#footnote-1)^


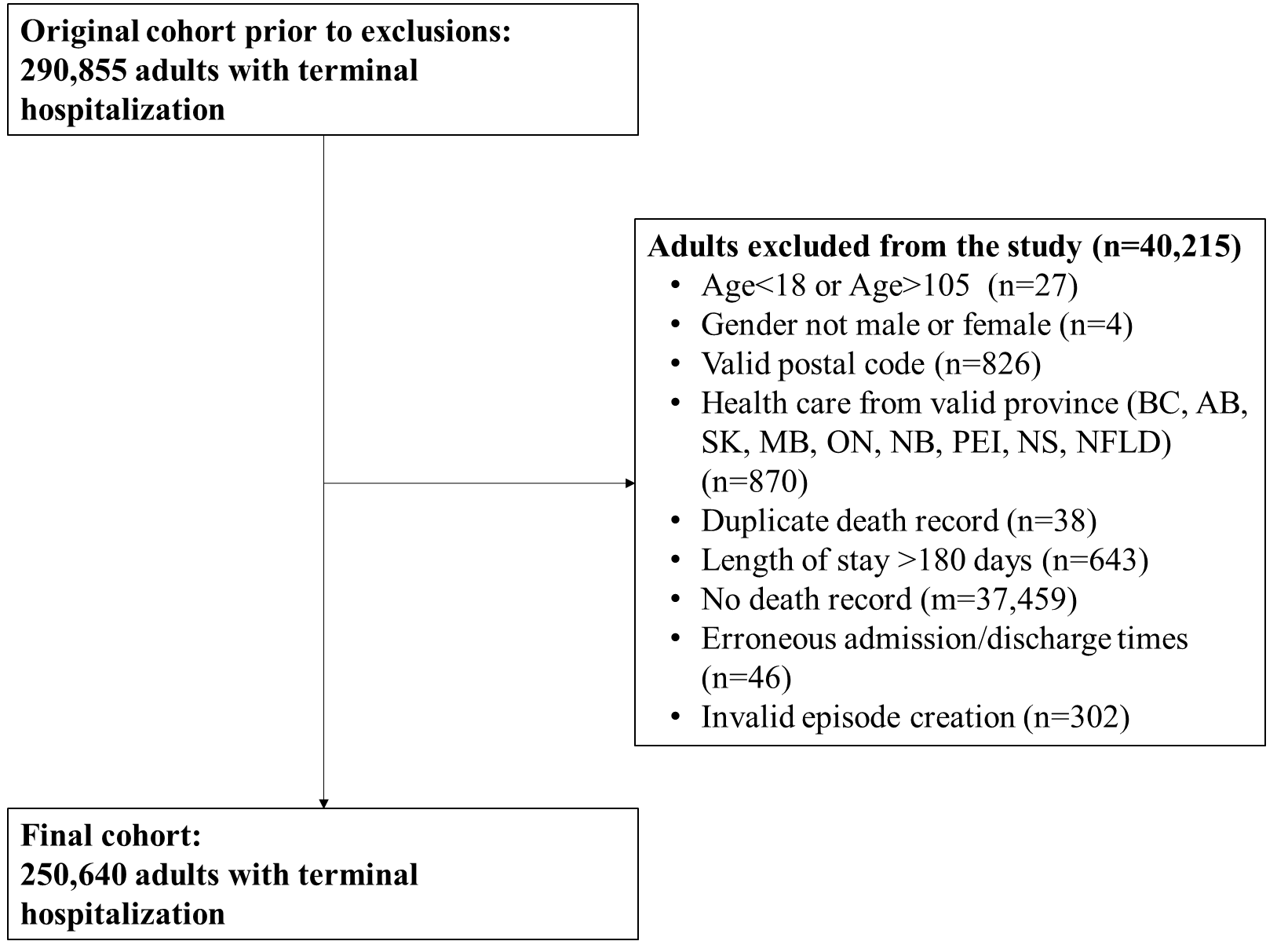


Table 1: Median/IQR cost per day in each level of palliative involvement and hospital length of stay.

| **Category** | | **No involvement median(IQR)**  **(n=95,450)** | **Low involvement median(IQR)**  **(n=94,849)** | **Medium-high involvement median(IQR)**  **(n=60,341)** |
| --- | --- | --- | --- | --- |
| Hospital length of stay (days) | 0-2 | 1291  (868, 2055) | 1134  (846, 1738) | 688  (569, 1049) |
|  | 3-7 | 1119  (921, 1801) | 1060  (914, 1488) | 728  (681, 838) |
|  | 8-19 | 1155  (905, 1867) | 1025  (873, 1444) | 729  (674, 841) |
|  | 20+ | 1064  (815, 1732) | 972  (790, 1394) | 741  (662, 914) |

1. Two additional items that we excluded for but that did not generate any exclusions and impact our cohort size were missing cost data and previous admissions that were greater than 12 months prior to death. [↑](#footnote-ref-1)
